# Diagnostic Performance of Electromagnetic Navigation versus Virtual Navigation Bronchoscopy-Guided Biopsy for Pulmonary Lesions in a Single Institution: Potential Role of Artificial Intelligence for Navigation Planning

**DOI:** 10.3390/diagnostics13061124

**Published:** 2023-03-16

**Authors:** Yuan-Ming Tsai, Yen-Shou Kuo, Kuan-Hsun Lin, Ying-Yi Chen, Tsai-Wang Huang

**Affiliations:** Division of Thoracic Surgery, Department of Surgery, Tri-Service General Hospital, National Defense Medical Center, Taipei City 11490, Taiwan

**Keywords:** pulmonary lesion, electromagnetic navigation bronchoscopy, virtual navigation bronchoscopy, biopsy, accuracy, safety, artificial intelligence

## Abstract

Navigation bronchoscopy is an emerging technique used to evaluate pulmonary lesions. Using Veran’s SPiN electromagnetic navigation bronchoscopy (ENB) and Archimedes virtual bronchoscopy navigation (VBN), this study aimed to compare the accuracy and safety of these procedures for lung lesions and to identify potentially relevant knowledge for the application of artificial intelligence in interventional pulmonology in a single institute. This single-center, retrospective study compared the ENB and VBN results in patients with pulmonary lesions unsuitable for biopsy via percutaneous transthoracic needle biopsy methods. A total of 35 patients who underwent navigation bronchoscopy for pulmonary lesion diagnosis were enrolled. Nineteen patients were stratified in the ENB group, and sixteen were in the VBN group. The mean age of this cohort was 67.6 ± 9.9 years. The mean distance of the lesion from the pleural surface was 16.1 ± 11.7 mm (range: 1.0–41.0 mm), and most lesions were a solid pattern (*n* = 33, 94.4%). There were 32 cases (91.4%) of pulmonary lesions with an air-bronchus sign. A statistically significant difference was found between pulmonary size and transparenchymal nodule access (*p* = 0.049 and 0.037, respectively). The navigation success rate was significantly higher in the VBN group (93.8% vs. 78.9%). Moreover, no procedure-related complications or mortality were noted. The radiographic characteristics, such as size or solid component, can affect the selection of the biopsy procedure, either ENB or VBN. Navigation bronchoscopy-guided biopsy demonstrated acceptable accuracy and a good safety profile in evaluating pulmonary lesions when the percutaneous approach was challenging or life threatening.

## 1. Introduction

Computed tomography (CT) of the chest is a cross-sectional evaluation and predominant medical imaging modality for assessing lung disease. With low-dose CT scan as a lung cancer screening tool, indeterminate pulmonary lesions with ground-glass opacities (GGOs) significantly increased [1]. Due to the fact of malignancy suspicion, diagnosing and treating small pulmonary nodules have become a focus for clinicians. The early detection and timely diagnosis of primary lung cancer are important due to the superior survival rate of stage I non-small cell lung cancer [2]. However, certainly, the diagnosis of a small pulmonary nodule is challenging using biopsy, bronchoscopy, and positron emission tomography CT [3].

Conventional bronchoscopy with biopsy has a low sensitivity in diagnosing these lesions and has a poor performance for lesions located at the outer third of the lung, especially for nodules of <2 cm in diameter [4]. The diagnosis rate of transbronchial biopsy using conventional bronchoscopy was approximately 18–62% [5]. In contrast, image-guided transthoracic needle aspiration is highly sensitive in diagnosing malignancy in these lesions, with a reported biopsy yield of 90%. Due to the high pneumothorax rate, tension pneumothorax, hemothorax, hemoptysis, hemorrhage, air embolism, and tumor seeding at the site of the biopsy route, it is unsuitable for patients with significant emphysema, blebs, lung vessels adjacent to lesions, and central lesions [6,7,8]. For these patients, a bronchoscopic-guided biopsy of pulmonary nodules may provide an alternative means with a lower risk of pneumothorax (1.5–3.1%) [9].

Navigation bronchoscopy is a minimally invasive procedure where physicians pass a bronchoscopy into the airways to allow for target lesion sampling. Electromagnetic navigation bronchoscopy (ENB; SPiN Thoracic Navigation System^®^, Veran Medical Technologies, Inc, St Louis, MO, USA) is an innovative technique using an electromagnetic tip-tracked biopsy instrument. Thereafter, a CT scan will be performed on the same day with respiratory sensors in place [10]. Several studies have reported diagnostic rates higher than conventional bronchoscopy [11,12]. The Archimedes virtual bronchoscopic navigation (VBN) system (Broncus Medical, San Jose, CA, USA) is an image-guided navigation system mainly applied to diagnose lung nodules. The system allows for vessel mapping, providing the best path to avoid blood vessels, which allows a combination of bronchoscopic transparenchymal nodule access (BTPNA), thereby reducing bleeding complications [13]. However, the comparison of the differences in the yield of ENB and VBN for diagnosing lung lesions is limited [12]. A meta-analysis showed diagnostic yields of 67% for ENB and 72% for VBN, with a substantial variation in the yield affected by the use of a guide sheath, ultrathin bronchoscope, and radial probe endobronchial ultrasound (R-EBUS) [14,15].

Artificial intelligence (AI) or machine-learning techniques have emerged as effective methods for integrating large-scale medical data for clinical practice in pulmonary diseases. However, few methods have significantly contributed to airway segmentation in bronchoscopy [16]. In general anesthesia, physicians introduce the bronchoscope through the endotracheal tube and directly reach the carina, which often cannot determine anatomical locations by tracing from the oral cavity to the deeper bronchi [17]. Although video bronchoscopy is a safe procedure and an important diagnostic tool for pulmonologists and chest surgeons, accurate airway navigation requires training to demonstrate competence for bronchoscopy in clinical settings [18,19]. However, the training environment can be somewhat disadvantageous, and the pandemic likely led to fewer learning opportunities for inexperienced surgeons and anesthesiologists. Previous reports showed that training experiences could lead to distinct differences in procedure lengths and complication rates [20]. Recently, researchers developed an AI model that accurately and quickly identifies and distinguishes anatomical locations using bronchoscopic images with a performance comparable with that of experienced pulmonologists [17]. ENB and VNB require prebronchoscopic CT scans to construct three-dimensional (3D) segmented pathways and additional software platforms. Therefore, AI-based applications only need bronchoscopic images to assist physicians directly by predicting the anatomical location of what he or she visualizes without any additional examinations to be performed.

Here, we report the safety, feasibility, and success rates of performing a diagnostic evaluation in a patient with pulmonary lesions utilizing Veran ENB and Archimedes VNB implementation to summarize the existing knowledge and indicate future steps required for the safe and practical application of AI tools for navigation planning.

## 2. Materials and Methods

**Figure 1:**
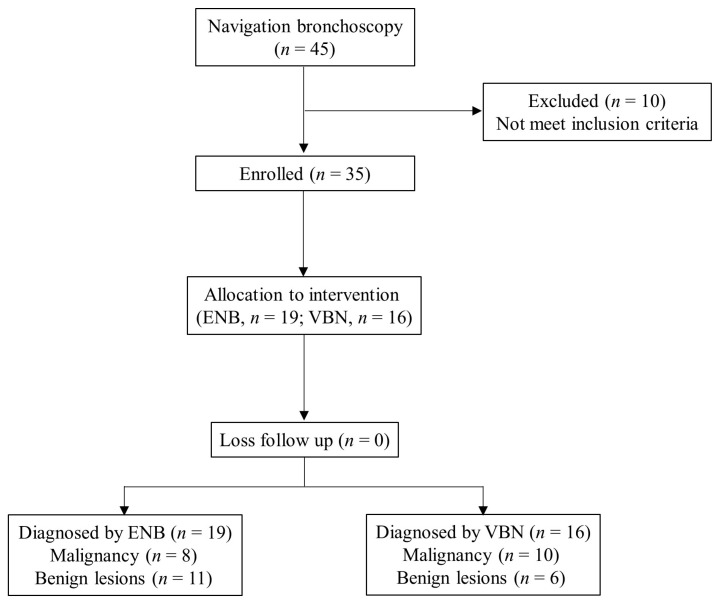
Flow diagram for the navigation bronchoscopy cases and results.

Thirty-five consecutive patients who underwent navigation bronchoscopy for pulmonary lesions unsuitable for biopsy via percutaneous transthoracic needle biopsy methods were retrospectively reviewed between November 2019 and November 2022. The inclusion criteria included age ≥ 18 years, pulmonary lesions of unknown origin, failure to have the tumor tissue pathologically diagnosed after evaluation in an interdisciplinary setting, and willingness to undergo the novel navigation bronchoscopy by both patients and their families. The exclusion criteria included inability to tolerate general anesthesia, presence of coagulopathies, long-term use of anticoagulants, abnormal platelet counts and function, history of airway bleeding, and incomplete medical records. A summary of the patient flow diagram is shown in Figure 1. Thirty-five patients satisfied the inclusion criteria and were retrospectively enrolled. Nineteen patients received the ENB for biopsy, and the other sixteen received the VBN. This study was approved by the Tri-Service General Hospital Institutional Review Board (no. C202205053).

### 2.1. Veran ENB Procedure

**Figure 2:**
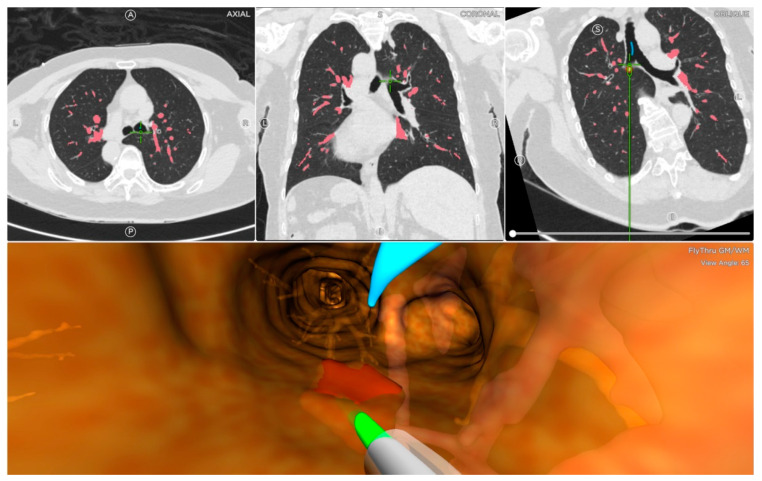
Veran electromagnetic navigational bronchoscopy leading to the lesion.

On the day of the ENB procedure, all patients underwent the inspiratory and expiratory 1 mm CT images with the placement of a navigational tracking pad (vPad2 Patient Tracker, INS-0050, Veran Medical, St Louis, MO, USA) on the anterior chest to create a virtual airway map (Veran Medical, St Louis, MO, USA). After the identification of the target lesion, the planning route to the pulmonary lesion was created by the software. Then, all patients were placed in a supine position and under general anesthesia. The peri-operative monitor was according to standard bronchoscopy monitoring protocols. Veran ENB was then used to locate, register, and navigate to the lung targets in accordance with the manufacturer’s instructions [21] (Figure 2). The routine procedure of ENB involved the only use of ENB without additional complementary tools, such as fluoroscopy and radial endobronchial ultrasound. Two chest surgeons performed the ENB procedures (Y. Y. C. and T. W. H., both had more than five years of experience with bronchoscopic procedures and a mean of 100 procedures·physician^−1^·yr^−1^). An electromagnetic tip-tracked biopsy instrument (Always-On Tip Tracked Forceps, 1.8 mm OD, Serrated Cup INS-0372, Veran Medical) was inserted via a bronchoscope with an outer diameter of 5.9 mm (Olympus BF-1TQ290) or 5.5 mm (Olympus BF-H190). The performing physician may use a needle aspiration instrument (Always-On Tip Tracked 21ga ANSO Cytology Needle INS-0392, Veran Medical). The biopsy instrument was navigated to the target lesion for the subsequent procedure. There were no other procedural techniques (e.g., additional bronchial washing, bronchoalveolar lavage, or cytology brushing).

### 2.2. Archimedes VNB Procedure

**Figure 3:**
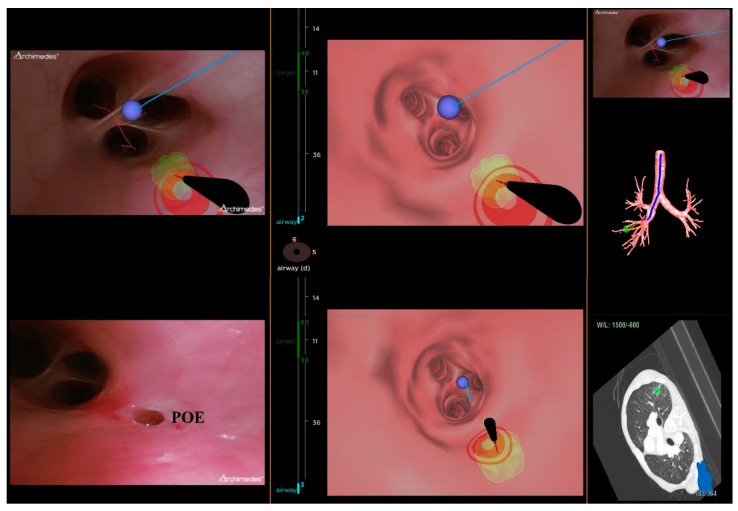
Archimedes virtual navigational bronchoscopy leading to the lesion.

All chest CT examinations for all patients were configured with 1 mm intervals in the Digital Imaging and Communications in Medicine format. The location board was placed on the patient’s back to create a localizing marker for the Archimedes VBN with possible transparenchymatous access. The CT images were uploaded into the Archimedes system to construct a 3D virtual airway leading to the pulmonary lesions. Archimedes can display different paths, determine the best path to avoid blood vessels, and measure the distance from the region of interest to the pleura [13]. After identifying the target lesion, the software can calculate and create the planning route (Figure 3). General anesthesia was inducted to all patients in the supine position. After, navigation with bronchoscopy (mentioned in the ENB procedure) was performed using the tools along the selected pathway to the target lesion of interest to utilize biopsy forceps and/or an 18-gauge needle to puncture the airway wall that was felt best suited to obtain a tissue sample. One chest surgeon performed the VNB procedures (Y. M. T. had more than five years of experience with bronchoscopic procedures and a mean of 100 procedures·yr^−1^). In the Archimedes Access Kit, there was an 18 ga FleXNeedle, sheath, and dilation balloon that could be utilized for the BTPNA, which was performed for 7 cases in our retrospective review. The BTPNA was performed with a robot-supported C-arm cone-beam CT, which featured 3D imaging using Syngo DynaCT Large Volume (Artis Pheno; Siemens Healthcare GmbH, Forchheim, Germany) to provide a real-time image model to mark the pulmonary lesions. This entailed locating the point of entry (POE) in the bronchus wall with/without balloon dilation, where a radiopaque sheath or biopsy forceps was introduced through the lung parenchyma to access the target lesion [15]. On average, approximately 3–4 samples were obtained using forceps in the Archimedes VNB procedure. The evaluation of the pathological biopsy specimens was then performed in both the ENB and VNB procedures. During and after surgery, data on the complications and management were documented. All patients received a postprocedural chest film within 2 h postoperatively to evaluate the possible postoperative complications. The postoperative period was uneventful.

### 2.3. Statical Analysis

The categorical variables were summarized by counts and percentages. The continuous variables were summarized by the mean, standard deviation, median, minimum, and maximum. The Pearson’s chi-squared or Fisher’s exact test was used to compare the categorical variables. For the comparison of the continuous variables, a Student’s *t*-test was used. The significant variables in the univariate analyses and those deemed clinically significant factors were included in the logistic regression analyses. All calculations were performed using Statistical Product and Service Solutions (version 22, SPSS Inc., Chicago, IL, USA), and a two-sided *p* < 0.05 was considered statistically significant.

## 3. Results

Thirty-five patients who underwent navigation bronchoscopy were included. Table 1 summarizes the patients’ clinical characteristics and pulmonary lesions. The mean age of the study population was 67.6 years (range: 48–85 years). There were 18 males (51.4%) and 17 females (48.6%) in this study. The mean body mass index was 23.2 kg/m^2^ (16.6–31.6 kg/m^2^). Among these patients, 13 (37.1%) had a smoking history. The mean pack-years was 32.2 (range: 10–75). The average entire procedure time was 54.8 ± 30.7 min (range: 13–127 min). Nineteen (54.3%) patients received the Veran ENB, and the other sixteen (45.7%) received the Archimedes VBN for the diagnosis. Based on the radiological findings, the mean size of the pulmonary lesion was 36.3 ± 21.9 mm (range: 11–128 mm), and the mean distance from the pleural surface to the lesion in its shortest path distance was 16.1 ± 11.7 mm (range: 1–41 mm). The tumors had a right lower lung lobe predominance (*n* = 12, 34.3%), followed by the right upper lobe (*n* = 11, 31.4%), left upper lobe (*n* = 6, 17.1%), right middle lobe (*n* = 4, 11.4%), and left lower lobe (*n* = 2, 5.7%). Most of the tumors were a solid pattern (*n* = 33, 94.3%). Additionally, most tumors in this cohort were positive for the air-bronchus sign (*n* = 32, 91.4%). The majority of the final histopathology results revealed a malignancy (*n* = 18, 51.4%). Among the 17 patients diagnosed with benign lesions, the majority were found to have benign inflammation (*n* = 14, 82.3%), followed by chronic bronchitis (*n* = 2, 11.8%) and lung granuloma (*n* = 1, 5.9%). No pneumothorax, hemorrhage, or tracheal injury occurred during the navigation bronchoscopy-guided biopsy. No procedure-related death was recorded.

Next, a subgroup analysis was conducted to compare the two navigation systems for biopsy (Table 2). There was no intergroup difference with regard to the patient characteristics: age (*p* = 0.194), sex ratio (*p* = 0.130), body mass index (*p* = 0.575), smoking history (*p* = 0.968), and mean pack-years (*p* = 0.788). When comparing the radiographic characteristics, the Veran ENB with tumor biopsy was mainly applied for larger pulmonary lesions compared with the Archimedes VBN (36.6 ± 16.5 vs. 27.6 ± 8.7 mm, *p* = 0.049). For transbronchial navigation, the Veran ENB needs a longer procedure time compared to the Archimedes VBN, but the difference was not significant (45.2 ± 17.6 vs. 38.33 ± 13.8 min, *p* = 0.313). Undoubtedly, the Archimedes VBN with BTPNA requires a longer procedure time (45.2 ± 17.6 vs. 66.8 ± 38.7 min, *p* = 0.037). No significant difference between the groups was found regarding tumor location (*p* = 0.301), distance from the pleura (*p* = 0.322), presence of the solid pattern (*p* = 0.181), and air-bronchus sign (*p* = 0.653). Malignancy, confirmed via histopathology, was predominant in the Archimedes VBN group (*n* = 10, 62.5%).

The Veran ENB was diagnostic in 89.5% (17/19) of the patients with a bronchus sign on the CT imaging, whereas the Archimedes VBN was performed in 93.8% (15/16) of the patients. The ENB sensitivity and specificity were 80.0% (8/10) and 77.8% (7/9), respectively, with a positive predictive value of 80.0% (8/10) and a negative predictive value of 77.8% (7/9). For smaller lung lesions, the Archimedes VBN had an improved success rate compared with the ENB (93.8% vs. 78.9%). The VBN sensitivity and specificity were 90.0% (9/10) and 100.0% (6/6), respectively, with a positive predictive value of 90.0% (9/10) and a negative predictive value of 85.7% (6/7) (Table 3).

## 4. Discussion

With the advancement of interventional pulmonology, several navigational bronchoscopic tools have been utilized to diagnose pulmonary nodules. Although the diagnostic yield of different bronchoscopic modalities has been widely studied, a limited study compared the diagnostic capabilities of ENB and VBN for pulmonary lesions [12]. Since the first human study performed in 2005, the introduction of ENB has allowed physicians to localize lung lesions, followed by biopsies through a minimally invasive procedure [22]. Most studies have reported ENB data using the SuperDimension navigate system, with limited data on ENB using the Veran navigate system on the Asian population [23]. A novel Archimedes platform for transparenchymal nodule access was first reported in a human study in 2015 [13]. The Archimedes VBN platform integrates virtual maps via CT imaging and real-time bronchoscopic imaging, providing an unprecedent level of control for physicians during the interventional procedure. A 3D airway roadmap reconstructed from CT scans to identify the target lesion is crucial and is a prerequisite for the procedure. In the current study, VBN improved the success rate with smaller lesions compared with ENB (93.8% for 27.6 ± 8.7 mm tumors and 78.9% for 36.6 ± 16.5 mm tumors). We observed a significant difference in the lesion size biopsied with ENB or VBN: 36.6 ± 16.5 mm (range: 11–80 mm) and 27.6 ± 8.7 mm (range: 14–41 mm), respectively (*p* = 0.049). The larger size of the pulmonary lesions in the ENB group could potentially reflect differences in the operator experience, as larger lesions may be easier to target and obtain a specimen. This could introduce confounding if the operator experience is also associated with success rate or complications. Interestingly, we found that in the ENB group, larger lesions had a lower success rate. One possible explanation for this disparity between the two groups may be due to the lack of real-time positioning with fluoroscopy in the ENB group. The utilization of intraprocedural CT has been shown to improve the diagnostic accuracy of bronchoscopic techniques in the diagnosis of pulmonary lesions [24,25,26,27,28]. CT-derived augmented fluoroscopy may provide integrated tracking, which would help operators identify otherwise invisible lesions during navigation bronchoscopic procedures.

The use of a singular or a combination of methods can influence the diagnostic yield from 38.5% to 80%, depending on the lesion location, size, bronchus sign, and use of an ultrathin bronchoscope [24,25,26,27]. Although GGOs or small lesions are not easily visible under fluoroscopy [28], marked lesions on cone-beam CT data could provide integrated tracking, which helps the operators identify lesions on the fluoroscopy image. In our study, the range of the distance of the lesions from the pleura was 1–41 mm. Performing a transthoracic needle biopsy in pulmonary masses only a few millimeters away from the chest wall can be technically challenging. It may increase the risk of complications, such as pneumothorax or bleeding. Furthermore, for frail and elderly patients, who could not be appropriately positioned for various reasons, and patients with lesions judged inaccessible in an ipsilateral-dependent position and the crowding of the ribs, a bronchoscopy-guided biopsy may be a less invasive and better-tolerated procedure than transthoracic needle biopsy. Navigation bronchoscopy can be performed under conscious sedation or general anesthesia, depending on the patient’s condition and preference. The lesions had a right lower and upper lobe predominance. In comparison to the results of other similar series, the results were consistent [23,29]. Although the CT bronchus sign was predominant in both groups, the relatively lower success rate in the Veran ENB was noted, indicating a quality issue of the preprocedure CT scan and registration algorithm to reconstruct airway anatomy [12,30]. Real-time imaging enabled operators to immediately realize the bronchial pathways and directions through the correlation between the Archimedes VBN route and the target lesion.

With the continuous improvement in computer learning and the accumulation of navigation bronchoscopy-related data, the application of AI in anatomical locations among the carina and both main bronchi under random rotation and covering has made significant progress in clinical decision support systems with video bronchoscopy [17]. An accurate airway segmentation and safe POE are essential to reduce operators’ burden of preprocedural airway identification and avoid possible complications to achieve a successful diagnosis. This remains a challenging task due to the complex tree-like structures and imbalanced sizes of airway generations. AI is a powerful tool used during the procedure and recently has become a valuable tool in pulmonology and cytopathology [31,32]. It constantly learns from each interaction and improves its performance gradually. Recently, the AI component of the navigation and biopsy guidance system had improvements, which rendered diagnostic biopsy very reliable [32,33]. However, the morphological complexity and imbalanced sizes, depth, and orientation in the bronchial tree can often be confusing, which makes it challenging to identify airway segmentation and tumor location for inexperienced bronchoscopist or chest surgeons. Recently, AI models have been developed; however, it needs to extract richer features during data preparation and preprocessing, followed by model training and evaluation and performance comparison with human experts [17]. Although a deep-learning-based pipeline (NaviAirway) utilized human-vision-inspired iterative training and a semi-supervised training approach to achieve higher accuracy and robustness, more data and comprehensive design are needed [16]. Currently, the high labeling costs for medical image processing, different training strategies for airway features with high or low bronchial generations, and model interpretability to increase its trustworthiness are still challenges for physicians in clinical applications.

In a review of the literature, the rate of incidentally detected lung nodules was approximately 2.1% via low-dose chest CT screening, and 95% comprised lung adenocarcinoma. Approximately 98% of patients received curative surgical resection as initial treatment [33]. Navigation bronchoscopy has been implemented increasingly by thoracic surgeons in preoperative marking procedures, followed by sublobar resection [25]. An AI model only requires video bronchoscopic or CT images without any additional examinations [34]. Moreover, the predicted anatomical location and possible POE can be overlaid in the virtual road map. The combination of an AI model in navigation bronchoscopy-guided preoperative marking methods and fused fluoroscopy can assist in the localization of small lesions, even in GGOs and in early-stage lung cancer, to minimize the risk of missing lesions or the conversion to unnecessary lobectomy or thoracotomy, which in turn translates into better control of the procedure and high diagnostic yield in the future.

### Limitations

There are some limitations to this study. First, this is a retrospective single institute study conducted by thoracic surgeons. All patients were referred to our division for a definitive diagnosis to aid in the decision regarding treatment by a pulmonologist or oncologist. The selection criteria for the ENB- or VBN-guided biopsy may vary depending on the surgeon’s experience, clinical setting, and the individual patient’s medical history and condition. Most patients could not undergo ENB due to the presence of vascular structures near the target lung lesions. The decision to use the ENB- or VBN-guided biopsy was made on a case review and discussion at multidisciplinary team meetings. Therefore, selection bias may influence the validity of the study. Second, the sample size is small, and some clinical information, such as the duration of the biopsy and number of biopsies used in each procedure, was not mentioned in the chart review. Third, two operators performed the ENB procedure and only one operator performed the VBN, which might hamper the generalizability of the results. It is possible that the experienced surgeon introduced bias in this study, which may eliminate the learning curve and undermine the experience of different surgeons.

## 5. Conclusions

Among available lung biopsy procedures, navigation bronchoscopy is safe and superior in assessing pulmonary lesions. Our study showed that the Archimedes Navigation System is a clinically feasible alternative to ENB at a single thoracic surgery division. The adoption of cone-beam CT may increase the navigation success rate, irrespective of the presence of the CT bronchus sign. Combination modalities and developments in AI may guide clinicians in challenging clinical scenarios, improving the quality of bronchoscopy training and the future of therapeutic management to achieve personalized medicine.

## Figures and Tables

**Table 1:** Characteristics of the patients and pulmonary lesions.

Characteristic	Value
Total	35
Age (years) mean (range)	67.6 ± 9.9 (48–85)
Sex (*n*, %)	
Male	18 (51.4%)
Female	17 (48.6%)
Body mass index	23.2 ± 3.6 (16.6–31.6)
Smoking history	13 (37.1%)
Pack-years mean (range)	32.2 ± 19.5 (10–75)
Procedure time (min)	54.8 ± 30.4 (13–127)
Lesion size (mm) mean (range)	36.3 ± 21.9 (11–128)
Location (*n*, %)	
Right upper lobe	11 (31.4%)
Right middle lobe	4 (11.4%)
Right lower lobe	12 (34.3%)
Left upper lobe	6 (17.1%)
Left lower lobe	2 (5.7%)
Distance from pleura (mm) mean (range)	16.1 ± 11.7 (1–41)
Radiographic characteristics (*n*, %)	
Ground glass opacity	0 (0.0%)
Part-solid	2 (5.7%)
Solid	33 (94.3%)
Air-bronchus present	32 (91.4%)
Histopathology	
Benign diagnosis	17 (48.6%)
Cancer diagnosis	18 (51.4%)

**Table 2:** Comparison of the two approaches.

Variable	ENB (*n* = 19)	VBN (*n* = 16)	*p*-Value
Age	69.6 ± 10.1	61.5 ± 9.7	0.194
Sex (Male)	12 (63.2%)	6 (37.5%)	0.130
Body mass index	23.5 ± 3.7	22.8 ± 3.5	0.575
Smoking history	7 (36.8%)	6 (37.5%)	0.968
Pack-years	30.7 ± 20.1	33.8 ± 20.6	0.788
Size (mm)	36.6 ± 16.5 (11–80)	27.6 ± 8.7 (14–41)	0.049
Procedure time (min)	45.2 ± 17.6 (13–83)	38.33 ± 13.8 (16–56) ^a^	0.313
		66.8 ± 38.7 (16–127) ^b^	0.037
Location (*n*, %)			0.301
Right upper lobe	5 (26.3%)	6 (37.5%)	
Right middle lobe	2 (10.5%)	2 (12.5%)	
Right lower lobe	7 (36.8%)	5 (31.3%)	
Left upper lobe	5 (26.3%)	1 (6.3%)	
Left lower lobe	0 (0.0%)	2 (12.5%)	
Distance from pleura (mm)	14.3 ± 12.4	18.3 ± 10.9	0.322
Radiographic characteristics (*n*, %)			0.181
Ground glass opacity	0 (0.0%)	0 (0.0%)	
Part-solid	2 (10.5%)	0 (0.0%)	
Solid	17 (89.5%)	16 (100.0%)	
Air-bronchus present	17 (89.5%)	15 (93.8%)	0.653
Histopathology			0.154
Benign diagnosis	11 (57.9%)	6 (37.5%)	
Cancer diagnosis	8 (42.1%)	10 (62.5%)	

^a^ Transbronchial navigation. ^b^ Combined bronchoscopic transparenchymal nodule access.

**Table 3:** Sensitivity and specificity of ENB versus VBN.

Variable	Sensitivity	Specificity	Positive Predictive Value	Negative Predictive Value
ENB	80.0% (8/10)	77.8% (7/9)	80.0% (8/10)	77.8% (7/9)
VBN	90.0% (9/10)	100.0% (6/6)	90.0% (9/10)	85.7% (6/7)

ENB: electromagnetic navigation bronchoscopy; VBN: virtual bronchoscopy navigation.

## Data Availability

All of the study’s data are reported in this article.
